# Fracture Occurrence Within FRAX-Defined High-Risk Myasthenia Gravis: An Exploratory Stratification by Age and Activities of Daily Living

**DOI:** 10.3390/jcm15020672

**Published:** 2026-01-14

**Authors:** Takafumi Uchi, Shingo Konno

**Affiliations:** Department of Neurology, Toho University Ohashi Medical Center, 2-22-36 Ohashi Meguro-Ku, Tokyo 153-8515, Japan; takafumi.uchi@med.toho-u.ac.jp

**Keywords:** myasthenia gravis, FRAX, osteoporotic fractures, activities of daily living, bone mineral density, falls

## Abstract

**Background/Objectives**: Patients with myasthenia gravis (MG) are at increased risk of osteoporotic fractures due to long-term oral corticosteroid use and disease-related muscle weakness. FRAX^®^ estimates 10-year fracture probability but does not incorporate falls or MG-specific functional impairment. To explore heterogeneity of fracture occurrence within MG patients classified as high risk by FRAX major osteoporotic fracture (MOF) probability. **Methods**: In a single-center retrospective cohort of 68 MG patients assessed in 2012, FRAX MOF with femoral neck BMD was calculable in 54 patients; the 29 patients with FRAX MOF ≥ 9.0% (the median of these 54 patients) comprised the high-FRAX cohort. Patients were stratified by the cohort medians of age (67 years) and MG-ADL (2 points) into four strata (HH, HL, LH, LL). This median-based stratification was exploratory and not intended as a clinically meaningful threshold. The primary outcome was time to first MOF (up to 10 years). We compared fracture occurrence using both proportions and Kaplan–Meier analyses (log-rank test) and performed exploratory univariable Cox models for selected predictors. No multivariable confounder adjustment was performed. **Results**: Eight of twenty-nine patients (27.6%) experienced an MOF. The proportions with MOF were HH 25.0%, HL 40.0%, LH 57.1%, and LL 0.0% (global *p* = 0.068). Kaplan–Meier curves differed across strata (log-rank *p* = 0.03), with separation most evident between LH and LL. For univariable Cox analyses, age was associated with shorter time to MOF (hazard ratio [HR] 1.13 per year, *p* = 0.041), and baseline difficulty rising from a chair (MG-ADL item) was associated with higher hazard rates (HR 3.45, *p* = 0.048). **Conclusions**: In this small, selected high-FRAX MG cohort, fracture events appeared to cluster in patients with impaired ADL and fall-related MG-ADL abnormalities, whereas FRAX values remained strongly age-driven. These findings are exploratory and hypothesis-generating and should not be interpreted as evidence of FRAX miscalibration; confirmation in larger, prospectively followed cohorts is needed.

## 1. Introduction

Myasthenia gravis (MG) is a chronic autoimmune disorder characterized by fluctuating muscle weakness and fatigability. Advances in immunotherapy and critical care have improved survival, so long-term complications such as osteoporosis and fragility fractures have become increasingly important in clinical practice [1,2,3,4]. In MG, prolonged exposure to oral corticosteroids, reduced physical activity, and disease-related muscle weakness may converge to increase fracture risk beyond that expected from age and sex alone [2,3]. Early identification of patients at high risk of fracture is, therefore, an essential component of comprehensive MG care.

Fragility fractures have major consequences for patients with MG, including pain, loss of independence, and prolonged rehabilitation, and may compromise respiratory function in those with pre-existing neuromuscular weakness [2]. Fractures also increase healthcare utilization and cost, underscoring the need for fracture risk assessment tailored to MG. Bone mineral density (BMD) measurement by dual-energy X-ray absorptiometry provides important information but is not always readily available in all settings and does not fully capture fracture risk related to falls, frailty, and neuromuscular dysfunction [5].

The Fracture Risk Assessment Tool (FRAX^®^) is widely used to estimate 10-year probabilities of major osteoporotic fractures based on age, sex, body mass index, prior fracture history, glucocorticoid use, and other clinical risk factors, with or without BMD measurement [6]. FRAX is simple and accessible, and threshold-based intervention strategies using FRAX have been proposed in several guidelines [6,7]. However, FRAX does not explicitly incorporate falls, neuromuscular disorders, or disease-specific functional impairment, even though prior falls are known to further increase fracture risk [7]. FRAX is also strongly age-dependent, which may lead to underestimation of fracture risk in younger individuals who have substantial functional limitations or frequent falls despite relatively low chronological age [6,8]. These characteristics raise concerns about the adequacy of FRAX alone in populations such as patients with MG.

MG-specific functional assessments may complement FRAX in this context. The Myasthenia Gravis Activities of Daily Living (MG-ADL) scale is an eight-item questionnaire that captures symptom severity and its impact on daily function, including bulbar, respiratory, ocular, and limb-girdle domains [9]. Minimal symptom expression (MSE), defined as an MG-ADL score of 0 or 1, has been proposed as a practical treatment target in MG. Ocular symptoms (ptosis and diplopia) can impair depth perception and spatial orientation, whereas difficulty standing up without using the arms reflects proximal lower limb weakness and impaired postural transitions. Both types of impairment are closely related to postural instability and falls in neuromuscular disorders [10,11].

We previously reported that combining FRAX and MG-ADL improves the identification of MG patients at risk of osteoporotic fractures compared with FRAX alone, suggesting that MG-ADL provides information that is not captured by FRAX [12]. In parallel, large population-based studies have reported increased fracture risk in MG, including recent nationwide data from Korea [13]. Our group has also published related analyses from the same clinical cohort using different research questions and endpoints (e.g., long-term BMD changes, FRAX-based fracture risk assessment, and functional augmentation of FRAX) [14,15]. The present study differs by restricting the analysis to the FRAX-defined high-risk subset (FRAX MOF with BMD ≥ cohort median) and by specifically examining heterogeneity of time-to-fracture across age × MG-ADL strata, with an emphasis on fall-related MG-ADL items. This work is intended as a hypothesis-generating rather than a validation study of FRAX performance.

In this study, we focused on MG patients already classified as high risk by FRAX major osteoporotic fracture (MOF) probability and stratified them by age and MG-ADL. Specifically, we divided patients into four strata according to age (high versus low, based on the cohort median) and total MG-ADL score (impaired versus preserved, also based on the median). We then compared MOF occurrence during up to 10 years of follow-up, FRAX MOF values, and the distribution of fall-related MG-ADL items—ocular symptoms and the need to use the arms when standing up—across these strata. We hypothesized that, within FRAX high-risk MG patients, fractures would be more frequent in younger patients with impaired ADL and fall-related functional deficits, whereas younger patients who achieved or approximated MSE would have a lower fracture incidence despite being categorized as high risk by FRAX.

## 2. Methods

### 2.1. Study Design and Setting

This was a single-center, retrospective observational study of patients with MG who attended the Department of Neurology at Toho University Ohashi Medical Center in 2012 and were followed from baseline until death, last available clinical contact, or 10 years after baseline. No formal sample size calculation was performed because this was an exploratory analysis of an existing cohort.

### 2.2. Participants

**Figure 1:**
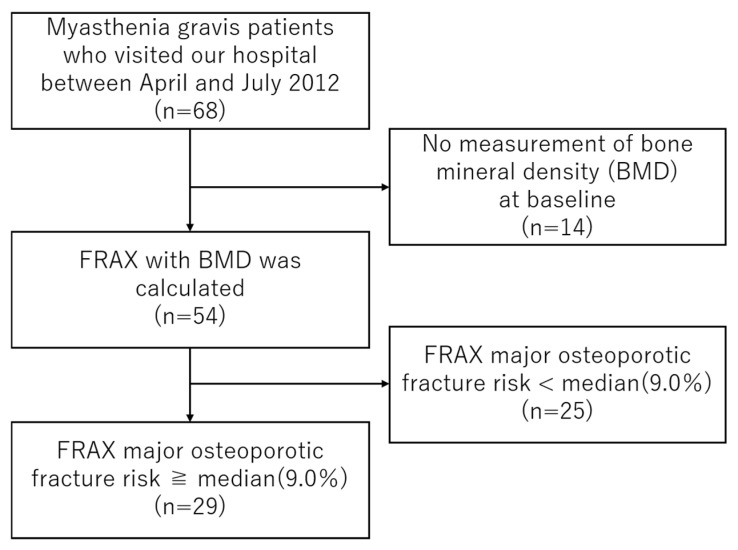
Patient selection flow in myasthenia gravis. Flowchart showing the selection of patients included in this study. Among 68 patients with myasthenia gravis (MG) who attended our hospital between April and July 2012, 14 had no bone mineral density (BMD) measurement at baseline and were excluded. FRAX major osteoporotic fracture (MOF) risk with BMD was calculated in 54 patients. Of these, 25 patients with FRAX MOF risk < median (9.0%) were excluded from the main analysis, leaving 29 patients in the high-FRAX cohort. Abbreviations: BMD, bone mineral density; FRAX, Fracture Risk Assessment Tool.

We initially identified 68 patients with MG who attended our clinic between April and July 2012. The flow of patient selection for the present analysis is shown in Figure 1.

MG was diagnosed according to established clinical criteria and supported by serological and electrophysiological findings, as appropriate. For each patient, we attempted to calculate the 10-year probability of a major osteoporotic fracture (MOF) using the FRAX^®^ tool (FRAX^®^ tool, University of Sheffield, Sheffield, UK; accessed on 1 September 2012) [16] with femoral neck BMD. Patients without available BMD measurements were excluded because FRAX with BMD could not be calculated. During follow-up, fractures clearly attributable to high-energy trauma or other non-osteoporotic causes were not counted as MOF outcomes; patients were not excluded solely because such non-osteoporotic fracture events occurred.

### 2.3. Clinical and Treatment Variables

For all patients in the high-FRAX cohort, we extracted baseline demographic, clinical, and treatment variables from medical records, including age, sex, disease duration, MG subtype, MG Foundation of America (MGFA) classification, MG-ADL score, and current and past use of oral corticosteroids and other immunosuppressants. We also recorded history of prior fragility fractures, concomitant osteoporosis treatments, and other FRAX clinical risk factors. These data were used to characterize the cohort and to compare baseline profiles across the age–ADL strata defined below.

### 2.4. Bone Mineral Density Measurement

Bone mineral density (BMD) was measured at baseline in 2012 by a dual-energy X-ray absorptiometry (DXA) system (GE Healthcare, Chicago, IL, USA) as part of routine clinical practice. Femoral neck BMD (g/cm^2^) was used as the DXA input for FRAX. In addition, lumbar spine and femoral neck BMD values were recorded for descriptive analyses, using standard acquisition and quality-control procedures at our institution.

### 2.5. FRAX Assessment

The 10-year probability of a major osteoporotic fracture (MOF)—defined in FRAX as a clinical vertebral, hip, forearm, or proximal humerus fracture—was estimated using the Japan-specific FRAX model with femoral neck BMD input. FRAX clinical risk factors were obtained from medical records; when a FRAX risk factor was not documented, it was coded as absent for calculation. Glucocorticoid exposure was coded according to standard FRAX definitions based on clinical documentation of oral corticosteroid therapy. Within the 54 patients with calculable FRAX MOF with BMD, we determined the cohort median FRAX MOF value (9.0%). Patients with FRAX MOF with BMD ≥ 9.0% were defined as the high-FRAX cohort for the present exploratory analyses.

### 2.6. MG-ADL Assessment

MG-related functional status at baseline was evaluated using the Myasthenia Gravis Activities of Daily Living (MG-ADL) scale [9]. This scale consists of eight items—speech, chewing, swallowing, breathing, brushing teeth/face washing, ability to stand up from a chair, ptosis, and diplopia—each scored from 0 (no symptoms) to 3 (most severe), yielding a total score ranging from 0 to 24. Minimal symptom expression (MSE) was defined as a total MG-ADL score of 0 or 1. In addition to the total score, the individual item scores were recorded. Because of their potential relevance to falls, particular attention was paid to three items: ptosis, diplopia, and the need to use the arms when rising from a chair. “Fall-related MG-ADL impairment” was defined as having a non-zero score in at least one of these three items at baseline. Other composite MG outcome measures, such as the MG Composite, capture overlapping constructs but are less widely used in routine clinical practice than MG-ADL [17].

### 2.7. Age–ADL Stratification

To investigate the combined impact of age and functional impairment within the high-FRAX cohort, we stratified the 29 patients according to the cohort medians of age and total MG-ADL score. In this cohort, the median age at baseline was 67 years and the median total MG-ADL score was 2 points. Using these cut-offs, we defined four strata. Patients with an age of 67 years or older and an MG-ADL score of 2 or higher were classified into the high-age/high-MG-ADL (HH) group (n = 8). Those with an age of 67 years or older and an MG-ADL score below 2 were assigned to the high-age/low-MG-ADL (HL) group (n = 5). Patients younger than 67 years with an MG-ADL score of 2 or higher were classified as the low-age/high-MG-ADL (LH) group (n = 7), and those younger than 67 years with an MG-ADL score below 2 formed the low-age/low-MG-ADL (LL) group (n = 9).

These median splits were chosen pragmatically to create four groups of comparable size in a small cohort and to allow an initial, exploratory description (with no intended clinical significance of the cut-offs) of different age–ADL profiles within the FRAX high-risk category. The cut-offs are therefore data-driven and not intended as externally validated clinical thresholds; dichotomizing continuous variables may introduce misclassification and loss of information, particularly when scores cluster at the low end. Because the median MG-ADL score in this cohort was 2, the low-MG-ADL category (MG-ADL < 2) mainly comprised patients with MG-ADL scores of 0 or 1 and thus roughly corresponded to minimal symptom expression (MSE).

### 2.8. Outcomes

The primary outcome of interest was the occurrence of a major osteoporotic fracture during follow-up after baseline. In accordance with the FRAX definition, a MOF was defined as a low-trauma fragility fracture involving the vertebral body (clinical vertebral fracture), proximal femur (hip fracture), distal forearm, or proximal humerus. Fracture events were identified from medical records, including imaging reports, outpatient notes, and discharge summaries. Only fractures consistent with osteoporosis, such as those occurring after a fall from standing height or less, were counted as MOF. Fractures clearly attributable to high-energy trauma, such as road traffic accidents, or to malignant bone disease, were excluded.

Patients were followed for up to 10 years after baseline. Because follow-up duration differed between strata, we recorded the length of follow-up for each patient and reported it by group. For the primary analysis, MOF was treated as a binary outcome (at least one MOF vs. none) during observed follow-up of up to 10 years.

Secondary outcomes included the distribution of FRAX MOF with BMD values across the four age–ADL strata and the presence of fall-related MG-ADL impairment at baseline in each stratum, as defined above. These outcomes were used to explore the relationships between age, FRAX-estimated fracture risk, functional status, and observed fracture incidence.

### 2.9. Statistical Analysis

Continuous variables were summarized as medians with interquartile ranges (IQR) because of the small sample size and the possibility of non-normal distributions. Categorical variables were described as counts and percentages. To compare baseline characteristics across the four age–ADL strata (HH, HL, LH, LL), we used the Kruskal–Wallis test for continuous variables and the chi-square test or Fisher’s exact test, as appropriate, for categorical variables. When overall group differences in continuous variables were significant, we performed exploratory pairwise comparisons using the Mann–Whitney U test with Bonferroni correction, as indicated in the tables.

For the primary outcome, we calculated, in each stratum, the proportion of patients who experienced at least one MOF during follow-up. Differences in MOF proportions among the four groups were examined using the chi-square test or Fisher’s exact test, depending on cell counts. For descriptive purposes, we also calculated the unadjusted risk difference and 95% confidence interval for MOF incidence between the LH and LL groups, which showed the largest contrast in proportions. FRAX MOF with BMD values were compared across strata using the Kruskal–Wallis test.

All statistical tests were two-sided. For descriptive group comparisons, a *p*-value < 0.05 was considered statistically significant. For exploratory pairwise comparisons following an overall test, we applied Bonferroni correction as indicated in the tables; however, given multiple outcomes and subgroup contrasts, the risk of type I error remains, and findings should be interpreted cautiously. Because follow-up duration differed across strata, we additionally evaluated time to first MOF using Kaplan–Meier curves and compared strata using the log-rank test. To explore time-to-event associations with selected baseline factors, we performed univariable Cox proportional hazards models (reported as hazard ratios with 95% confidence intervals). Given the small number of fracture events, multivariable confounder adjustment was not performed; the Cox analyses are presented as hypothesis-generating. All analyses were performed with EZR (version 1.54; Saitama Medical Center, Jichi Medical University, Saitama, Japan) [18], which is a graphical user interface for R (version 4.0.3; R Foundation for Statistical Computing, Vienna, Austria).

## 3. Results

### 3.1. Baseline Characteristics of the High-FRAX Cohort

Baseline demographic and clinical characteristics of these four strata are summarized in Table 1.

Among the 68 patients with MG who attended our clinic in 2012, 54 had BMD measurements that allowed calculation of FRAX MOF with BMD, and 29 of these 54 patients (53.7%) had FRAX MOF with BMD ≥ 9.0% and were included in the high-FRAX cohort. Within this cohort, the median age at baseline was 67 years, and the median MG-ADL total score was 2 points. When stratified by age and MG-ADL into four groups (HH, HL, LH, LL), the distribution of demographic and MG-related characteristics was broadly similar across strata, although age-related differences were evident, and some traditional FRAX risk factors appeared more common in older strata.

### 3.2. FRAX MOF Values Across Age–ADL Strata

FRAX MOF with BMD values in the high-FRAX cohort showed a skewed distribution toward higher probabilities, as expected from the selection criteria (Table 2).

Median FRAX MOF with BMD values in each stratum were as follows: HH: 18.5 [13.5–26.5] %, HL: 29.0 [21.0–30.0] %, LH: 14.0 [10.2–26.5] %, and LL: 10.0 [9.6–12.0] %. By contrast, when grouped by age, FRAX MOF with BMD tended to be higher in older patients than in younger patients, regardless of MG-ADL status, confirming the strong influence of chronological age on FRAX estimates. By design, all patients in this analysis were above the median FRAX MOF threshold; however, these values illustrate the range of FRAX-estimated risk within the high-FRAX cohort and highlight the contrast with actual fracture occurrence in each stratum.

### 3.3. Major Osteoporotic Fracture Incidence

**Figure 2:**
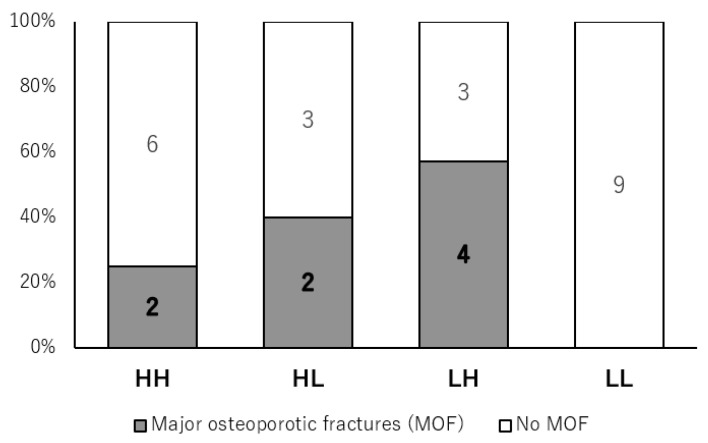
Ten-year incidence of major osteoporotic fracture by age–ADL stratum. Stacked bar chart showing the proportion of patients with (grey) and without (white) major osteoporotic fractures (MOF) during follow-up of up to 10 years in four age–ADL strata: high age/high MG-ADL (HH), high age/low MG-ADL (HL), low age/high MG-ADL (LH), and low age/low MG-ADL (LL). Numbers within each segment denote the number of patients in each outcome category. The overall difference among strata was not statistically significant (global *p* = 0.068).

During follow-up of up to 10 years after baseline, 8 of the 29 patients (27.6%) in the high-FRAX cohort experienced at least one MOF. The distribution of fracture events across the four age–ADL strata is shown in Table 2 and Figure 2. The global *p*-value (*p* = 0.068) reflects an overall comparison of MOF proportions across strata and does not account for censoring or unequal follow-up duration; therefore, we additionally report time-to-event analyses using Kaplan–Meier curves and the log-rank test (Supplementary Figure S1).

Because follow-up duration differed across strata, we additionally examined time to first MOF using Kaplan–Meier curves (Supplementary Figure S1). Survival curves differed among groups (log-rank test, *p* = 0.03), with separation most evident between the LH and LL strata. In exploratory univariable Cox models (Supplementary Table S1), age was associated with time to MOF (HR 1.13 per year, *p* = 0.041) and baseline difficulty rising from a chair was associated with higher hazard (HR 3.45, *p* = 0.048), whereas ocular items were not statistically significant.

MOF incidence in each group was HH, 2/8 patients (25.0%); HL, 2/5 patients (40.0%); LH, 4/7 patients (57.1%); and LL, 0/9 patients (0.0%). Median follow-up durations were 9.6 years in the HH group, 3.0 years in the HL group, 10.9 years in the LH group, and 11.0 years in the LL group.

The overall difference in MOF incidence among the four strata did not reach statistical significance (global *p* = 0.068). When the LH and LL groups were compared directly, the unadjusted risk difference in MOF incidence was 57 percentage points, with a 95% confidence interval of approximately 20% to 94%. Thus, although the contrast between these two strata appears large and is statistically significant in this pairwise comparison, the estimate remains imprecise because of the small sample sizes, and the non-significant global *p* value indicates that clear separation across all four strata cannot be established.

Taken together, these results indicate that, within the FRAX-defined high-risk MG cohort, fracture events in this small sample were concentrated in the low-age/high-ADL stratum, whereas no fractures occurred in the low-age/low-ADL stratum. However, the limited number of events, differences in follow-up duration, and wide confidence intervals mean that these apparent gradients should be interpreted cautiously.

### 3.4. Fall-Related MG-ADL Impairments Across Strata

**Figure 3:**
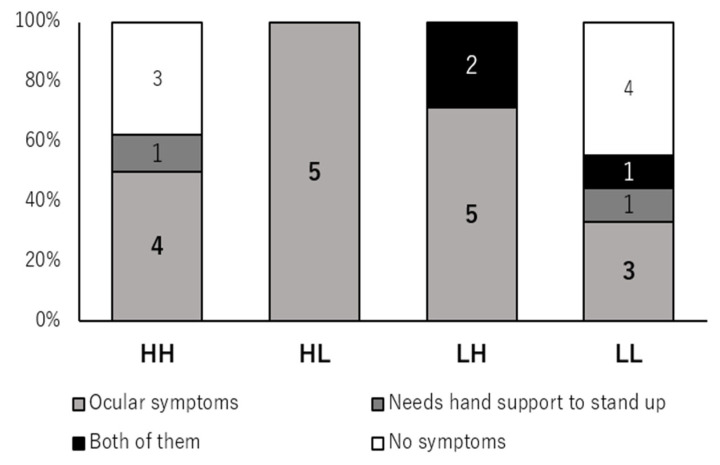
Distribution of fall-related MG-ADL impairments according to age–ADL strata. Stacked 100% bar chart showing the distribution of fall-related MG-ADL items in the four age–ADL strata: high age/high MG-ADL (HH), high age/low MG-ADL (HL), low age/high MG-ADL (LH), and low age/low MG-ADL (LL). Each bar represents all patients in that stratum and is divided into four mutually exclusive categories: ocular symptoms only (ptosis and/or diplopia; light grey), difficulty standing up from a chair requiring hand support only (dark grey), both ocular symptoms and difficulty standing up (black), and neither of these impairments (white). From bottom to top, segments correspond to ocular symptoms only, standing difficulty only, and both impairments, with the white segment indicating no fall-related MG-ADL impairment. Numbers within each segment indicate the number of patients. All patients in the LH group had at least one fall-related MG-ADL impairment, whereas many patients in the LL group had none and were close to minimal symptom expression.

We next examined the distribution of fall-related MG-ADL impairments—defined as a non-zero score in at least one of ptosis, diplopia, or the need to use the arms when rising from a chair—across the four age–ADL strata (Table 1 and Figure 3).

Fall-related MG-ADL impairments were more common in the HL and LH strata than in the LL stratum, and the LH group had the highest proportion of patients with at least one such impairment. In contrast, fall-related MG-ADL impairments were rare in the LL group, whose members largely achieved or approximated MSE, whereas the HH group showed a mixed pattern with some patients having ocular or proximal lower limb impairment and others not.

The co-occurrence of numerically higher MOF incidence and frequent fall-related MG-ADL impairment in the LH group suggests that fall-related functional deficits may contribute to fracture risk among younger MG patients who are already classified as high risk by FRAX. Conversely, the relatively low fracture incidence in the LL group, despite being in the same high-FRAX category and sharing traditional risk factors, aligns with the preservation of fall-related functions and proximity to MSE in this subgroup.

## 4. Discussion

In this exploratory analysis restricted to MG patients classified as high risk by FRAX MOF with BMD, fracture occurrence varied across age–MG-ADL strata. Although the overall four-group comparison of MOF proportions did not reach conventional statistical significance (global *p* = 0.068), time-to-event analysis accounted for unequal follow-up and showed group differences (log-rank *p* = 0.03; Supplementary Figure S1). Given the small sample size and limited number of events, these results should be interpreted as hypothesis-generating.

Importantly, the present study does not evaluate FRAX calibration or discrimination and therefore cannot determine whether FRAX over- or underestimates risk in MG. Rather, within a selected high-FRAX subset, FRAX values remained strongly age driven, while fractures appeared to occur more frequently in strata with functional impairment and fall-related MG-ADL abnormalities. This pattern is consistent with the concept that disease-specific functional measures may capture dimensions of fracture risk not explicitly represented in FRAX [6,7,8,12].

In exploratory univariable Cox models, baseline difficulty rising from a chair was associated with time to MOF (HR 3.45), whereas ocular items were not statistically significant (Supplementary Table S1). These findings are biologically plausible because impaired sit-to-stand reflects proximal lower-limb weakness and impaired postural transitions, which can increase fall propensity [19,20,21,22]; moreover, falls and physical performance measures have been shown to predict fractures independently of FRAX probability [23,24]. At the same time, the between-group differences in total MG-ADL were modest (e.g., median 2 vs. 1 points for LH vs. LL in this cohort), below commonly cited minimal clinically important differences (~2 points), underscoring the need for cautious interpretation and external validation.

From a clinical and research perspective, these data suggest that, among MG patients who already meet a relatively high-FRAX definition within a given cohort, additional stratification by functional status—particularly fall-relevant domains—may help generate hypotheses about which patients are most fracture-prone. However, the present results are not sufficient to recommend changes in clinical practice, and prospective studies with systematic fall recording, larger event counts, and formal predictive-performance analyses are required.

Several limitations warrant emphasis. This was a single-center retrospective study with potential selection and information bias, and the cohort was predominantly female, which may limit generalizability. The age and MG-ADL cut-offs were data-driven medians, and dichotomization of continuous variables may have introduced misclassification and reduced statistical information. Event counts were small, restricting confounder adjustment and increasing uncertainty in time-to-event estimates. In addition, because multiple analyses have been performed using the same underlying clinical cohort across publications, there is a risk of inflated type I error; we therefore frame this work as exploratory and provide de-identified data elements needed to reproduce the current analyses as Supplementary Materials.

Other limitations include the lack of systematically recorded falls, which precluded direct evaluation of falls as a mediator between MG functional impairment and fractures. In routine clinical documentation of this retrospective cohort, fall events were not systematically ascertained, precluding reliable chart-based capture. We therefore explored fall-relevant functional proxies using MG-ADL items, including the need to use the arms when rising from a chair. This approach is consistent with chair-stand-based fall-risk screening frameworks and prior reports linking chair-stand performance to falls and fall-related injury [19,20,21,22]. These proxies cannot replace direct fall ascertainment; prospective studies with systematic fall recording are warranted, and the use of a relative FRAX threshold (median within the BMD-available cohort) rather than a guideline-defined high-risk cut-off. Accordingly, the term “high risk” in this manuscript refers to the upper half of this cohort’s FRAX distribution and may not correspond to treatment thresholds in other settings.

## 5. Conclusions

In this exploratory single-center study of MG patients selected as relatively high risk by FRAX MOF with BMD, fracture events were not evenly distributed across age–MG-ADL strata, and time-to-event curves suggested heterogeneity in MOF risk.

Within this selected cohort, fall-relevant functional impairment (e.g., difficulty rising from a chair) was associated with time to MOF in exploratory Cox models, whereas FRAX values remained strongly age-driven. These observations are hypothesis-generating and support further prospective evaluation of integrated, MG-specific functional measures alongside FRAX, rather than indicating a definitive shortcoming of FRAX itself.

Clinically, our results support an integrated approach to fracture risk assessment in MG that combines FRAX with MG-ADL, with particular attention to fall-related domains, to identify patients who should be prioritized for preventive interventions. Given the small sample size and retrospective design, these conclusions should be regarded as hypothesis-generating. Prospective, multi-center studies are required to validate whether incorporating MG-ADL into risk stratification improves prediction of fractures and guides more effective, targeted prevention strategies in patients with MG.

## Figures and Tables

**Table 1:** Baseline characteristics by age–ADL group in myasthenia gravis.

Factor	Category/Unit	HH (n = 8)	HL (n = 5)	LH (n = 7)	LL (n = 9)	*p* Value
Demographics						
Sex	Female	7 (87.5)	5 (100.0)	7 (100.0)	7 (77.8)	0.425
Age	years	73.5 [70.8, 76.5] ‡**	81.0 [75.0, 85.0] §¶	66.0 [64.0, 66.5]	64.0 [60.0, 64.0]	<0.001
Age at MG onset	years	61.0 [47.5, 68.8]	72.0 [48.0, 79.0]	37.0 [31.0, 46.0]	49.0 [38.0, 50.0]	0.083
Disease duration	years	12.0 [4.8, 24.0]	6.0 [4.0, 26.0]	27.0 [19.0, 31.5]	13.0 [10.0, 26.0]	0.397
MG-related characteristics						
MG type	OMG	1 (12.5)	2 (40.0)	0 (0.0)	3 (33.3)	0.544
	gEOMG	2 (25.0)	2 (40.0)	4 (57.1)	4 (44.4)	
	gLOMG	3 (37.5)	1 (20.0)	1 (14.3)	0 (0.0)	
	TAMG	1 (12.5)	0 (0.0)	1 (14.3)	2 (22.2)	
	Ab-negative MG	1 (12.5)	0 (0.0)	1 (14.3)	0 (0.0)	
Worst-ever MGFA class	I	1 (12.5)	3 (60.0)	0 (0.0)	4 (44.4)	0.229
	II	1 (12.5)	1 (20.0)	3 (42.9)	3 (33.3)	
	III	3 (37.5)	0 (0.0)	2 (28.6)	0 (0.0)	
	IV	2 (25.0)	0 (0.0)	1 (14.3)	0 (0.0)	
	V	1 (12.5)	1 (20.0)	1 (14.3)	2 (22.2)	
MGFA post-intervention status	CSR	0 (0.0)	0 (0.0)	0 (0.0)	1 (11.1)	0.285
	PR	0 (0.0)	0 (0.0)	0 (0.0)	1 (11.1)	
	MM	2 (25.0)	3 (60.0)	0 (0.0)	4 (44.4)	
	I	6 (75.0)	2 (40.0)	6 (85.7)	3 (33.3)	
	U	0 (0.0)	0 (0.0)	1 (14.3)	0 (0.0)	
MG-ADL total score	points	2.5 [2.0, 6.0] †‡	1.0 [0.0, 1.0]	2.0 [1.0, 3.0]	1.0 [1.0, 1.0]	0.009
Speech		0.0 [0.0, 0.0]	0.0 [0.0, 0.0]	0.0 [0.0, 0.0]	0.0 [0.0, 0.0]	0.453
Chewing		0.0 [0.0, 0.0]	0.0 [0.0, 0.0]	0.0 [0.0, 0.0]	0.0 [0.0, 0.0]	0.453
Swallowing		0.0 [0.0, 0.2]	0.0 [0.0, 0.0]	0.0 [0.0, 0.0]	0.0 [0.0, 0.0]	0.142
Breathing		0.0 [0.0, 0.0]	0.0 [0.0, 0.0]	0.0 [0.0, 0.0]	0.0 [0.0, 0.0]	0.453
Brushing teeth/face washing		0.5 [0.0, 1.0]	0.0 [0.0, 0.0]	0.0 [0.0, 0.5]	0.0 [0.0, 0.0]	0.240
Needs hand support to stand up		0.5 [0.0, 1.0] ‡	0.0 [0.0, 0.0]	0.5 [0.0, 1.0]	0.0 [0.0, 0.0]	0.038
Ptosis		0.5 [0.0, 1.0]	1.0 [1.0, 2.0]	1.0 [1.0, 1.0]	0.0 [0.0, 1.0]	0.058
Diplopia		1.0 [0.0, 1.2]	0.0 [0.0, 0.0]	0.5 [0.0, 1.0]	0.0 [0.0, 0.0]	0.032
Sum of ocular and standing-up items		1.0 [0.0, 1.0]	2.0 [2.0, 2.0]	2.0 [1.5, 3.0] #	1.0 [0.0, 1.0]	0.038
QMG score	points	5.5 [4.8, 8.5] ‡	2.0 [2.0, 4.0]	7.0 [6.0, 9.5] #	2.0 [1.0, 3.0]	0.001
MG composite score	points	5.5 [3.5, 7.2] ‡	0.0 [0.0, 2.0]	5.0 [4.0, 7.5] #	0.0 [0.0, 1.0]	0.002
MG-QOL15 total score	points	27.0 [21.8, 31.5] †	3.0 [3.0, 11.0]	16.0 [13.5, 29.0]	14.5 [6.2, 15.2]	0.028
Treatment-related factors						
Current PSL dose	mg/day	2.0 [0.0, 2.2]	5.8 [1.1, 11.2]	2.0 [1.0, 5.5]	2.0 [0.0, 4.5]	0.824
Maximum PSL dose	mg/day	45.0 [37.5, 48.8]	12.5 [8.1, 23.8]	50.0 [40.0, 50.0]	35.0 [21.2, 50.0]	0.235
Duration of PSL therapy	years	4.8 [3.5, 10.4]	14.5 [2.8, 29.5]	10.0 [3.9, 16.5]	4.0 [2.6, 9.0]	0.897
Cumulative PSL dose in the last year	mg	895 [46, 1076]	697 [49, 3965]	916 [381, 1213]	981 [104, 1863]	0.957
Total cumulative PSL dose	mg	2241 [1849–2526]	300 [131–1260]	2300 [1824–2599]	2151 [1739–2309]	0.100
Calcineurin inhibitor use	n (%)	6 (75.0)	3 (60.0)	4 (57.1)	3 (33.3)	0.382
Tacrolimus dose	mg/day	3.0 [3.0, 3.0]	2.0 [1.0, 2.0]	3.0 [2.2, 3.2]	2.0 [0.0, 4.5]	0.824
Cyclosporine dose	mg/day	2.5 [2.2, 3.5]	NA	0.0 [0.0, 0.0]	2.5 [2.4, 2.5]	0.322

Note: Categorical variables are expressed as n (%). Continuous variables are expressed as median [interquartile range]. † *p* < 0.05 for HH vs. HL; ** *p* < 0.01 for HH vs. LH; ‡ *p* < 0.05 for HH vs. LL; § *p* < 0.05 for HL vs. LH; ¶ *p* < 0.05 for HL vs. LL; # *p* < 0.05 for LH vs. LL (Mann–Whitney U test with Bonferroni correction). Abbreviations: HH, HL, LH and LL indicate high age–high MG-ADL, high age–low MG-ADL, low age–high MG-ADL and low age–low MG-ADL groups, respectively; MG, myasthenia gravis; OMG, ocular myasthenia gravis; gEOMG, generalized early-onset myasthenia gravis; gLOMG, generalized late-onset myasthenia gravis; TAMG, thymoma-associated myasthenia gravis; Ab-negative MG, antibodies-negative myasthenia gravis; MGFA, Myasthenia Gravis Foundation of America; CSR, complete stable remission; PR, pharmacological remission; MM, minimal manifestations; I, improved; U, unchanged; MG-ADL, Myasthenia Gravis–Activities of Daily Living; QMG score, Quantitative Myasthenia Gravis score; MG-QOL15, 15-item Myasthenia Gravis Quality of Life scale; PSL, prednisolone; NA, not applicable.

**Table 2:** Bone status and FRAX-based fracture risk by age–ADL group in myasthenia gravis.

Factor	Category/Unit	HH (n = 8)	HL (n = 5)	LH (n = 7)	LL (n = 9)	*p* Value
Major osteoporotic fracture within 10 years	n (%)	2 (25.0)	2 (40.0)	4 (57.1)	0 (0.0)	0.068
Bone density, bone metabolism and FRAX						
Lumbar spine BMD	g/cm^2^	0.8 [0.7, 0.8]	0.6 [0.6, 0.6]	0.7 [0.6, 0.8]	0.8 [0.7, 0.9]	0.151
Femoral neck BMD	g/cm^2^	0.6 [0.5, 0.7] †	0.5 [0.4, 0.5] ¶	0.6 [0.5, 0.6]	0.6 [0.6, 0.7]	0.048
T-score (femoral neck)	SD	−1.6 [−2.1, −0.9]	−2.9 [−3.5, −2.6] ¶	−2.1 [−2.7, −1.8]	−1.6 [−2.3, −1.1]	0.013
Serum BAP	μg/L	11.0 [8.0, 15.5]	12.0 [11.0, 15.5]	13.0 [11.5, 13.5]	13.0 [12.0, 15.0]	0.899
Serum NTX	nmol BCE/L	14.8 [12.5, 21.4]	14.0 [9.5, 16.8] ¶	12.1 [11.3, 13.5]	13.7 [10.4, 14.4]	0.493
FRAX 10-yr hip fracture risk (with BMD)	%	6.2 [2.6, 9.8]	13.0 [8.5, 18.0] ¶	2.6 [1.7, 8.3]	2.0 [0.7, 3.0]	0.016
FRAX 10-yr major osteoporotic fracture risk (with BMD)	%	18.5 [13.5, 26.5] ‡	29.0 [21.0, 30.0]	14.0 [10.2, 26.5]	10.0 [9.6, 12.0]	0.006
Bisphosphonate use	n (%)	8 (100.0)	4 (80.0)	4 (80.0)	7 (100.0)	0.265
Fracture risk factors for FRAX						
Previous fragility fracture	n (%)	0 (0.0)	0 (0.0)	1 (14.3)	0 (0.0)	0.354
Parental hip fracture	n (%)	1 (12.5)	0 (0.0)	1 (14.3)	1 (11.1)	0.864
Current smoking	n (%)	0 (0.0)	0 (0.0)	0 (0.0)	1 (11.1)	0.512
Glucocorticoid use	n (%)	6 (75.0)	3 (60.0)	6 (85.7)	7 (77.8)	0.782
Rheumatoid arthritis	n (%)	2 (25.0)	0 (0.0)	0 (0.0)	0 (0.0)	0.131
Secondary osteoporosis	n (%)	0 (0.0)	0 (0.0)	0 (0.0)	0 (0.0)	NA
Alcohol intake	n (%)	0 (0.0)	0 (0.0)	0 (0.0)	0 (0.0)	NA
Follow-up						
Follow-up duration	years	9.6 [6.0, 11.0]	3.0 [2.8, 4.2]	10.9 [9.8, 10.9]	11.0 [10.9, 11.0]	0.035

NOTE: Categorical variables are expressed as n (%). Continuous variables are expressed as median [interquartile range]. † *p* < 0.05 for HH vs. HL; ‡ *p* < 0.05 for HH vs. LL; ¶ *p* < 0.05 for HL vs. LL (Mann–Whitney U test with Bonferroni correction). Abbreviations: HH, HL, LH and LL indicate high age–high MG-ADL, high age–low MG-ADL, low age–high MG-ADL and low age–low MG-ADL groups, respectively; ADL, activities of daily living; BMD, bone mineral density; FRAX, Fracture Risk Assessment Tool; BAP, bone-specific alkaline phosphatase; NTX, cross-linked N-telopeptide of type I collagen expressed as bone collagen equivalents (BCE); SD, standard deviation; NA, not applicable.

## Data Availability

The Cox model output is provided as Supplementary Table S1. De-identified participant-level data required to reproduce the analyses are not publicly available due to institutional and legal restrictions but can be made available by the corresponding author upon reasonable request.

## Supplementary Materials

The following supporting information can be downloaded at: https://www.mdpi.com/article/10.3390/jcm15020672/s1, Figure S1. Kaplan–Meier curves for time to first major osteoporotic fracture (MOF) by combined risk groups (HH, HL, LH, LL). The y-axis indicates the cumulative proportion without MOF (“No MOF rate”), and tick marks denote censored observations. Numbers at risk are shown below the plot. Survival curves differed significantly among groups (log-rank test, *p* = 0.03), with separation most evident between the LH and LL groups; Table S1. Univariable Cox proportional hazards models for time to first MOF.

## References

[1] Gilhus N.E. (2015). Myasthenia gravis. Lancet Neurol..

[2] Yeh J.-H., Chen H.-J., Chen Y.-K., Chiu H.-C., Kao C.-H. (2014). Increased risk of osteoporosis in patients with myasthenia gravis: A population-based cohort study. Neurology.

[3] Konno S., Suzuki S., Masuda M., Nagane Y., Tsuda E., Murai H., Imai T., Fujioka T., Suzuki N., Utsugisawa K. (2015). Association between glucocorticoid-induced osteoporosis and myasthenia gravis: A cross-sectional study. PLoS ONE.

[4] Binks S., Vincent A., Palace J. (2016). Myasthenia gravis—A clinical-immunological update. J. Neurol..

[5] Varacallo M., Seaman J., Jialal I. (2025). Osteoporosis. StatPearls.

[6] Kanis J.A., Johnell O., Oden A., Johansson H., McCloskey E.V. (2008). FRAX™ and the assessment of fracture probability in men and women from the UK. Osteoporos. Int..

[7] Kanis J.A., Johansson H., Harvey N.C., Lorentzon M., Liu E., Vandenput L., Morin S., Leslie W.D., McCloskey E.V. (2023). Adjusting conventional FRAX estimates of fracture probability according to the number of prior falls in the preceding year. Osteoporos. Int..

[8] Silverman S.L., Calderon A.D. (2010). The utility and limitations of FRAX: A US perspective. Curr. Osteoporos. Rep..

[9] Muppidi S. (2011). The myasthenia gravis activities of daily living profile: Still a relevant outcome measure. Muscle Nerve.

[10] Horlings C.G.C., van Engelen B.G.M., Allum J.H.J., Bloem B.R. (2008). A weak balance: The contribution of muscle weakness to postural instability and falls. Nat. Clin. Pract. Neurol..

[11] Pieterse A.J., Luttikhold T.B., de Laat K., Bloem B.R., van Engelen B.G.M., Munneke M. (2006). Falls in patients with neuromuscular disorders. J. Neurol. Sci..

[12] Konno S., Uchi T., Kihara H., Sugimoto H. (2025). Functional Status Enhances the FRAX^®^ Prediction of Fractures in Myasthenia Gravis: A 10-Year Cohort Study. J. Clin. Med..

[13] Park H.S., Kim K., Yu M.H., Shin H.Y., Rhee Y., Kim S.W., Hong N. (2024). Risk of fracture in patients with myasthenia gravis: A nationwide cohort study in Korea. J. Bone Miner. Res..

[14] Konno S., Uchi T., Kihara H., Sugimoto H. (2024). Ten-year fracture risk in Japanese patients with myasthenia gravis: A comprehensive assessment using the fracture risk assessment tool. J. Neurol. Sci..

[15] Konno S., Uchi T., Kihara H., Sugimoto H. (2024). Long-Term Bone Density Changes and Fracture Risk in Myasthenia Gravis: Implications for FRAX^®^ Tool Application. Healthcare.

[16] Fujiwara S., Nakamura T., Orimo H., Hosoi T., Gorai I., Oden A., Johansson H., Jönsson B., Kanis J.A. (2008). Development and application of a Japanese model of the WHO fracture risk assessment tool (FRAX™). Osteoporos. Int..

[17] Burns T.M., Conaway M.R., Cutter G.R., Sanders D.B., MG Composite and MG-QOL15 Study Group (2008). Construction of an efficient evaluative instrument for myasthenia gravis: The MG Composite. Muscle Nerve Off. J. Am. Assoc. Electrodiagn. Med..

[18] Kanda Y. (2013). Investigation of the freely available easy-to-use software ‘EZR’ for medical statistics. Bone Marrow Transplant..

[19] Centers for Disease Control and Prevention (CDC) STEADI: Assessment—30-Second Chair Stand. https://www.cdc.gov/steadi/media/pdfs/STEADI-Assessment-30Sec-508.pdf.

[20] Applebaum E.V., Breton D., Feng Z.W., Ta A.T., Walsh K., Chassé K., Robbins S.M. (2017). Modified 30-second Sit to Stand test predicts falls in a cohort of institutionalized older veterans. PLoS ONE.

[21] Shea C.A., Ward R.E., Welch S.A., Kiely D.K., Goldstein R., Bean J.F. (2018). Inability to Perform the Repeated Chair Stand Task Predicts Fall-Related Injury in Older Primary Care Patients. Am. J. Phys. Med. Rehabil..

[22] Buatois S., Miljkovic D., Manckoundia P., Gueguen R., Miget P., Vançon G. (2008). Five times sit to stand test is a predictor of recurrent falls in healthy community-living subjects aged 65 and older. J. Am. Geriatr. Soc..

[23] Harvey N.C., Odén A., Orwoll E., Lapidus J., Kwok T., Karlsson M.K., Rosengren B., Johansson H., McCloskey E.V., Kanis J.A. (2018). Falls predict fractures independently of FRAX probability: A meta-analysis of the Osteoporotic Fractures in Men (MrOS) Study. J. Bone Miner. Res..

[24] Harvey N.C., Odén A., Orwoll E., Lapidus J., Kwok T., Karlsson M.K., Rosengren B., Johansson H., McCloskey E.V., Kanis J.A. (2018). Measures of physical performance and muscle strength as predictors of fracture risk independent of FRAX, falls, and aBMD: A meta-analysis of the Osteoporotic Fractures in Men (MrOS) Study. J. Bone Miner. Res..

