# Supplementary material for: Fracture Occurrence Within FRAX-Defined High-Risk Myasthenia Gravis: An Exploratory Stratification by Age and Activities of Daily Living
Source: J Clin Med. 2026 Jan 14;15(2):672. doi: 10.3390/jcm15020672 (PMC12842129; doi:10.3390/jcm15020672)
Supplement: Supplementary file 1 [file jcm-15-00672-s001.zip › jcm-4057944-supplementary.pdf]

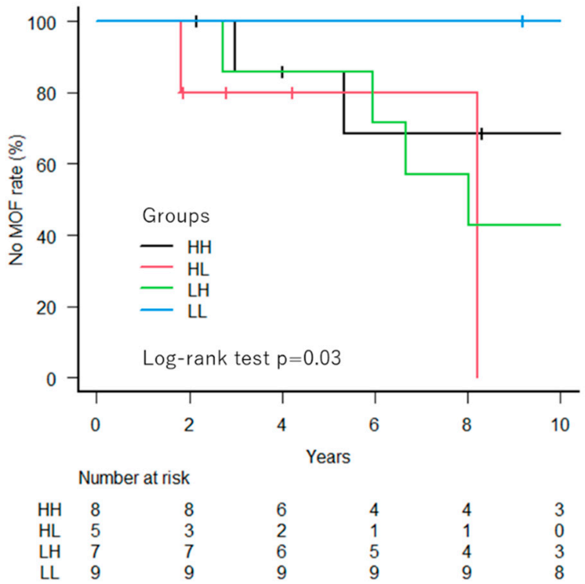

Supplementary Figure S1. Kaplan–Meier curves for time to first major osteoporotic fracture (MOF) by combined risk groups (HH, HL, LH, LL). The y-axis indicates the cumulative proportion without MOF (“No MOF rate”), and tick marks denote censored observations. Numbers at risk are shown below the plot. Survival curves differed significantly among groups (log-rank test, p = 0.03), with separation most evident between the LH and LL groups.

Factor	Hazard. Ratio	p.value
Age (year)	1.13 (1.00-1.27)	**0.04**
ADL scale (point)		
ptosis	0.84 (0.33-2.11)	0.71
diplopia	1.36 (0.45-4.15)	0.59
standing	3.45 (1.01-11.71)	**0.04**

Supplementary Table S1. Univariable Cox proportional hazards models for time to first MOF
